# Subclinical Hypothyroidism after ^131^I-Treatment of Graves’ Disease: A Risk Factor for Depression?

**DOI:** 10.1371/journal.pone.0154846

**Published:** 2016-05-02

**Authors:** Jing Yu, Ai-Juan Tian, Xin Yuan, Xiao-Xin Cheng

**Affiliations:** 1 Nuclear Medicine Division, Second Affiliated Hospital, Dalian Medical University, Dalian 116023, China; 2 Department of Cell Biology and the Liaoning Provincial Key Laboratory of Cancer Genomics and Epigenetics, College of Basic Medical Sciences, Dalian Medical University, Dalian 116044, China; University of Rochester, UNITED STATES

## Abstract

**Objectives:**

Although it is well accepted that there is a close relationship between hypothyroidism and depression, previous studies provided inconsistent or even opposite results in whether subclinical hypothyroidism (SCH) increased the risk of depression. One possible reason is that the etiology of SCH in these studies was not clearly distinguished. We therefore investigated the relationship between SCH resulting from ^131^I treatment of Graves’ disease and depression.

**Design And Methods:**

The incidence of depression among 95 patients with SCH and 121 euthyroid patients following ^131^I treatment of Graves’ disease was studied. The risk factors of depression were determined with multivariate logistic regression analysis. Thyroid hormone replacement therapy was performed in patients with thyroid-stimulating hormone (TSH) levels exceeding 10 mIU/L.

**Results:**

Patients with SCH had significantly higher Hamilton Depression Scale scores, serum TSH and thyroid peroxidase antibody (TPOAb) levels compared with euthyroid patients. Multivariate logistic regression analysis revealed SCH, Graves’ eye syndrome and high serum TPO antibody level as risk factors for depression. L-thyroxine treatment is beneficial for SCH patients with serum TSH levels exceeding 10 mIU/L.

**Conclusions:**

The results of the present study demonstrated that SCH is prevalent among ^131^I treated Graves’ patients. SCH might increase the risk of developing depression. L-thyroxine replacement therapy helps to resolve depressive disorders in SCH patients with TSH > 10mIU/L. These data provide insight into the relationship between SCH and depression.

## Introduction

In the general population, the prevalence of clinical hypothyroidism is around 1% [1]. Its main presentations are decreased metabolic and sympathetic activities. Typical symptoms include chills, hypohidrosis, hand and foot swelling, joint pain, drowsiness, fatigue, depression, memory loss, weight gain, and sexual dysfunction. Clinical signs also include pale facial coloration, facial swelling, dry skin, sluggishness, hoarseness, decreased responsiveness, decreased heart rate, pretibial myxedema, and impaired cardiac function. Serum levels of free tri-iodothyronine and thyroxine (FT3 and FT4) are decreased and thyroid-stimulating hormone (also known as thyrotropin, TSH, or hTSH for human TSH) levels are elevated. Clinical hypothyroidism is a risk factor for depression [1–4].

Subclinical hypothyroidism (SCH) is defined by elevated TSH levels, FT_4_ concentration in serum within normal limits, and absence of clinical signs and symptoms. The prevalence of SCH in the general population is higher than that of clinical hypothyroidism and lacks specific signs and symptoms. According to the Chinese Thyroid Disease Treatment Guidelines, the overall incidence of SCH in the general population is between 4% and 10%, in the United States the incidence is between 4% and 8.5%, and in China between 0.91% and 6.05% [5]. The incidence of SCH increases with age and is higher in women than in men, and it is up to about 20% in women over the age of 60. SCH is associated with an elevated risk of clinical hypothyroidism and abnormalities of lipid metabolism [5].

Hypothyroidism is closely linked to depression [1–4]. Several pieces of evidence support this claim. Hypothyroidism and depression show similar clinical manifestations, including apathy, lethargy, and impaired learning and memory. Some patients with hypothyroidism, who mainly present with depressive symptoms, are misdiagnosed with depression. The incidence of depressive disorder among hypothyroidism patients is higher than in the general population. The incidence of hypothyroidism in patients with depressive disorder is also higher than in the general population [1–4,6]. Therapeutic use of thyroid hormone in patients with depressive disorder has been shown to relieve symptoms significantly [2,4].

The relationship between SCH and depressive disorder, however, is controversial. SCH has been shown to be a risk factor for depressive disorder [6–9]. However, two recent studies performed on large patient populations failed to confirm any increased incidence of depression among patients with SCH [10,11]. To date, no formal indications have been recommended for the psychopathological evaluation in SCH patients.

^131^I treatment is the primary treatment for diffuse goiter (Graves’ disease). The advantages include low recurrence rate, low liver toxicity, and minimal bone marrow suppression. ^131^I accumulates in thyroid cells and the β-radiation leads to shrinkage of thyroid follicles and reduction in thyroid hormones over time, which in turn suppress thyroid function. For most patients, the symptoms begin to resolve within two to three weeks of ^131^I treatment, peripheral thyroid hormone levels return to normal and manifestations of Graves’ disease resolve completely within two to three months,. The main side effect of ^131^I therapy is hypothyroidism. Within one year of ^131^I treatment, the incidence of hypothyroidism is 15% to 20%, which increases progressively at an annual rate of 2% to 3%. ^131^I treatment of Graves’ disease also increases the risk of SCH. The incidence of SCH among Graves’ patients following ^131^I treatment has rarely been reported, whether this group of patients is at high risk for depressive disorders is still unclear, and the therapeutic effect of thyroid hormone treatment in these SCH patients is controversial. Therefore, the purpose of this study was to determine the incidence of and the risk factors for depression in patients with Graves’ disease who developed SCH after ^131^I treatment, and to evaluate the therapeutic effect of thyroid hormone replacement in these patients.

## Material and Methods

### Study patients

Two hundred and sixteen patients with Graves’ disease were enrolled in this retrospective case-control study at the Dalian Medical University affiliated hospital No. 2. Ninty-five patients presented with SCH after receiving ^131^I therapy. The SCH group included 34 male and 61 female patients aged from 33 to 69 years (mean age 45.1 ± 5.3 years). The diagnosis of Graves’ disease was in accordance with the Chinese Thyroid Disease Treatment Guidelines [5]. As a control group, 121 Graves’ disease patients were enrolled without any symptoms of SCH after ^131^I therapy. This group included 48 male and 73 female patients whose age ranged from 31 to 65 years (mean age 46.5 ± 6.6 years). Serum thyroid hormone and TSH levels were within normal limits among patients in the control group.

The time of enrollment after diagnosis of Graves’ disease ranged between 6 months and 22 years. Diagnostic criteria for SCH were based on the Chinese Thyroid Disease Treatment Guidelines [5]. The criteria included a TSH concentration higher than the statistically determined upper limit of the reference value, FT_4_ concentrations in the serum within the normal range, and exclusion of other possible causes of increased serum TSH. The other possible causes include falsely elevated TSH values caused by serum TSH autoantibodies; low T_3_ syndrome, in which convalescent serum TSH is elevated to 5∼20 mIU/L, perhaps due to stress response; hypothalamic or pituitary dysfunction, 20% of which is associated with TSH elevation (5∼10 mIU/L); renal dysfunction, which may be associated with low levels of thyroid hormone-binding protein, reduced TSH metabolism, and excess intake of iodine; reduced adrenal function due to glucocorticoid deficiency; and cold stimulation exceeding nine months, which can lead to a 30∼50% increase in serum TSH.

None of the patients in either group had any history of other thyroid diseases or psychiatric disorders, and none had recently taken psychotropic drugs or medications affecting thyroid function. All patients signed an informed consent form. The study was approved by the ethics committee of the Dalian Medical University Affiliated Hospital No. 2.

### Evaluation of depression and SCH treatment

Patients were interviewed by physicians with extensive experience in clinical psychiatry. The diagnosis of depression was established based on the International Classification of Diseases (ICD-10). The presence of depressive symptoms was assessed by means of the HAMD (Hamilton Depression Scale). For SCH patients whose serum TSH levels exceeded 10 mIU/L, L-thyroxine treatment was administered (25 μg L-thyroxine oral per day). The HAMD evaluation was repeated six months after TSH concentrations were normalized.

### Serological analysis

Serum TSH and FT_4_ were measured with electrochemiluminescent immunoassay kit purchased from Roche Ltd (Shanghai, China). The sensitivity of the TSH measurement was 0.05 mIU/L. The inter-assay coefficient was 6.2% and the intra-assay coefficient was 9.8%. The normal range was 0.27∼4.20 mIU/L for TSH and 12.00∼22.00 pmol/L for FT_4_. Serum thyroid autoantibodies, including thyroid peroxidase antibodies (TPOAb) and thyrotrophin receptor antibodies (TRAb), were measured using radioimmunoassay kits obtained from the North Biotech Institute (Beijing, China). The reference level was 0.00∼34.00 IU/L for TPOAb and 0∼1.75 IU/L for TRAb.

### Statistical analysis

Data were analyzed using the Statistical Package for the Social Sciences (SPSS, v16, SPSS, Inc., Chicago, IL, USA). First, a simple descriptive statistics was conducted to determine the relationship between depression and each of the other variates. Chi-square analysis was performed for qualitative data and the independent-samples *t*-test was performed for quantitative data. Only variates that were significantly correlated with depression were chosen to be analyzed in the following steps. The association between depression in Graves’ patients after ^131^I treatment and the potential risk factors was examined using multiple logistic regression. Odds ratios (OR) for multivariate analysis and 95% confidence intervals (95% CI) were calculated. Values of *p* < 0.05 were considered to be statistically significant.

## Results

No significant differences were found with respect to age, gender, or serum FT_4_ levels between the SCH group and the control group with Graves’ disease. Significantly higher serum TSH levels were detected in the SCH group compared with the control group. The TPOAb and TRAb levels in both groups were elevated, and the TPOAb level of the SCH group was significantly higher compared with the control group. No difference in TRAb levels was observed between the two groups (Table 1).

**Table 1:** Clinical demographics of Graves’ disease patients.

	Control group (n = 121)	SCH group (n = 95)
Female (n,%)	73 (60.3)	61 (64.2)
Male (n,%)	48 (39.7)	34 (35.8)
Marrital status (n, %)		
Married	108 (89.3)	77 (81.1)
Unmarried	13 (10.7)	18 (18.9)
Eye syndrome (n, %)	11 (9.1)	22 (23.2) ^△^
Family history (n, %)	18 (14.9)	9 (9.5)
Age (years)	46.53 ± 6.61	45.12 ± 5.34
Education (years)	13.31 ± 3.16	14.65 ± 3.42
TSH (mIU/L)	2.64 ± 0.71	17.3 ± 0.85^△^
FT_4_ (pmol/L)	17.63 ± 3.72	14.63 ± 2.37
TPOAb (IU/L)	55.39 ± 8.64	84.75 ± 10.36*
TRAb (IU/L)	22.31 ± 5.13	27.25 ± 6.78
Heart rate	78.64 ± 8.51	75.83 ± 7.39
HAMD score	12.67 ± 3.82	22.71 ± 4.26*
Depression (n, %)		
Baseline	8 (6.6)	25 (26.3) ^△^
6-month	2 (1.7)	7 (7.4)

* *P* < 0.05

^△^ *P* < 0.01 compared with the control group.

Based on ICD-10 evaluation, twenty-five patients in the SCH group were diagnosed with depression (26.3%) after ^131^I treatment, while the symptoms of depression resolved in 18 patients after anti-depression treatment. Eight (6.6%) of the 121 control patients matched the diagnostic criteria for depression after ^131^I treatment of Graves’ disease. The HAMD score was significantly higher in the SCH group compared with the control group, and high serum TSH levels were related to a high HAMD score (*x*^2^ = 11.67, *p* < 0.01; Table 2).

**Table 2:** Relationship between HAMD score and TSH levels in Graves’ disease patients after ^131^I treatment.

TSH (mIU/L)	HAMD score ≤17	HAMD score >17
0.27∼4.20	69 (51.9%)	26 (31.3%)
4.21∼10.00	30 (22.6%)	25 (30.1%)
10.01∼20.0	17 (12.8%)	25 (30.1%)
>20.00	17 (12.8%)	10 (12.0%)
Total	133	83

*x*^2^ = 11.67, *p* < 0.01

Serum levels of TSH and TPOAb were found to be significantly higher in patients with depression compared with patients without depression. However, no significant differences were detected with respect to FT_4_ and TRAb levels (Table 3).

**Table 3:** Serum markers in patients with and without depression.

	Patients without depression (n = 183)	Patients with depression (n = 33)
TSH	4.83 ± 0.76 mIU/L	16.75 ± 4.96 mIU/L^△^
FT_4_	17.46 ± 3.95 pmol/L	14.83 ± 4.64 pmol/L
TPOAb	50.37 ± 9.83 IU/L	91.17 ± 11.21 IU/L^△^
TRAb	25.01 ± 6.54 IU/L	28.72 ± 7.36 IU/L

^△^*P* < 0.01 compared with patients without depression

The reference level for TPOAb was 0.00∼34.00 IU/L

**Fig 1:**
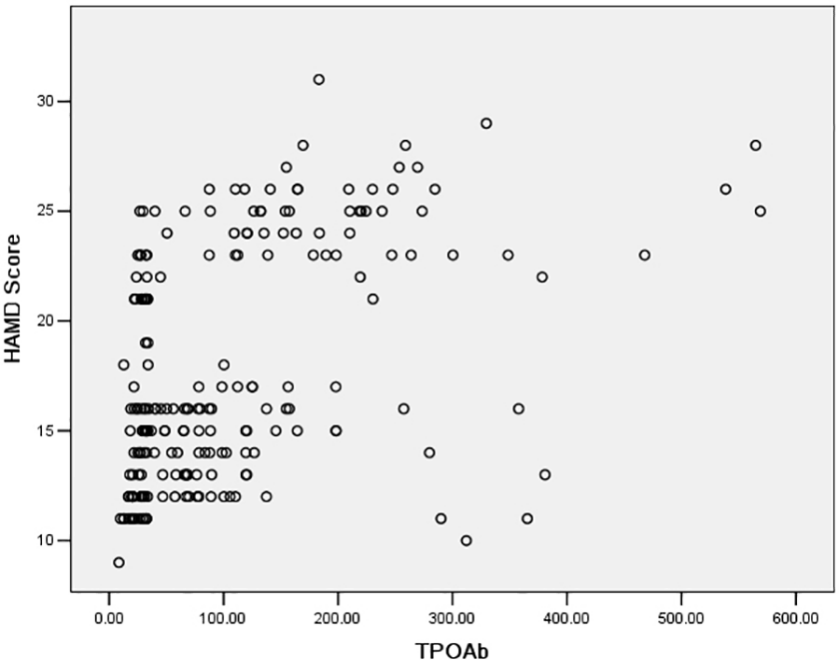
Scatter plot of correlation between HAMD score and TPOAb.

Initial statistics revealed that SCH, Graves’ eye syndrome, and serum level of TPOAb were significantly correlated with depression (Table 4, Fig 1). These potential risk factors of depression were then included in the following statistical analysis. Multivariate logistic regression analysis was performed to investigate the risk factors of depression in Graves’ patients after ^131^I treatment. The results revealed that SCH was associated with an 3.153-fold increased risk of depression. Additionally, Graves’ eye syndrome was accompanied by a 4.412-fold increased risk of depression. The high serum TPOAb levels were accompanied by a 1.862-fold increased risk of depression in Graves’ patients after ^131^I treatment (Table 5).

**Table 4:** Contingency table analysis of the correlation between TPOAb and HAMD score.

	HAMD score > 17	HAMD score ≤ 17
High TPOAb^a^	60	70
Normal TPOAb^b^	25	61

^a^ TPOAb > 34.00 IU/L

^b^ TPOAb ≤ 34.00 IU/L

95% CI: 1.172∼3.733, *P* = 0.012.

**Table 5:** Multivariate logistic regression analysis of risk factors of depression after ^131^I treatment of Graves’ disease.

	OR^a^	95% CI^b^	*P*-Value
SCH	3.153	1.204∼6.972	0.017
TPOAb	1.862	1.068∼2.490	0.024
Eye syndrome	4.412	1.267∼8.631	0.006

^a^ OR, odds ratio

^b^ CI, Confidence interval

In the SCH group, serum TSH levels were found to exceed 10 mIU/L in 66 patients, among whom 28 patients received L-thyroxine therapy. Patients diagnosed with depression underwent antidepressant therapy, and therefore the efficacy of L-thyroxine therapy could not be determined in this group. The initial dosage of L-thyroxine was 25 μg oral per day and serum TSH levels were measured monthly for six months. If serum TSH levels remained elevated, additional doses of 25 μg L-thyroxine were administered until serum TSH returned to normal. Depression was reassessed six months later using the HAMD, and significantly lower rates of depression were observed in SCH patients (*t* = 2.963, *p* < 0.01).

## Discussion

The results of the present study show a significantly higher prevalence of depression in patients with SCH following ^131^I treatment of Graves’ disease compared with patients without SCH. This result was consistent with previous studies in patients with SCH of other etiologies [6,7]. The relationship between SCH and depressive disorders is controversial. A study with a larger patient population (n = 1561) found that SCH was not a risk factor for depression [11]. In another study, Jorde et al. used the Beck Depression Inventory and found similar depression scores between individuals with SCH (n = 89) and unaffected patients (n = 154) [10]. However, Correia et al. reported significantly higher anxiety and depression scores in patients with SCH (n = 14) compared with unaffected patients (n = 19) [9]. In women with a history or a family history of thyroid disease, the prevalence of depression in those with SCH (9/15) was three times higher compared with unaffected patients (3/15) [7]. Chueire et al. reported that in SCH patients over 60, the prevalence of depression was as high as 49.7% (74/149) [6]. However, in another study among patients over 85, no correlation between serum TSH and depressive disorder was found [12]. Similarly, in a recent study among patients older than 65 years, no differences in depression rating scales were detected between SCH patients (n = 165) and unaffected individuals (n = 754) [13].

There are several possible reasons for these inconsistent findings. 1) The studies used different evaluation standards for depression. In the majority of studies, depression scales were developed internally. Depression rating scales, such as the Beck Depression Inventory, have not been standardized (i.e. ICD-10 or DSM-IV). 2) The study participants lack comparability between studies. Some studies use SCH as the criteria and do not distinguish between specific types of hypothyroidism, such as primary hypothyroidism, antithyroid drug-induced hypothyroidism, inflammation-induced hypothyroidism, or central hypothyroidism. Some studies enroll only specific types of individuals, such as the elderly or women. 3) Patients with SCH may have other, non-thyroid illnesses that may lead to depression, anxiety, or other psychiatric disorders. 4) Finally, several of the studies have relied on very small study populations.

The risk factors of depression among Graves’ patients treated with ^131^I have not been previously reported. The present study demonstrated that SCH, Grave’s eye syndrome, and high serum TPOAb levels increased the risk of depression following ^131^I treatment of Graves’ disease. All patients in both the SCH group and the control group were diagnosed with Graves’ disease before ^131^I treatment and had no history of other thyroid disease or significant non-thyroid disorder at the time of diagnosis. ICD-10 standards were used in the diagnosis of depression, which increased the reliability of these results. Since Graves’ disease is essentially an autoimmune disease, antithyroid antibodies may be present in the patients’ blood. In the present study, all Graves’ disease patients treated with ^131^I showed elevated serum levels of TPOAb and TRAb, and the TPOAb levels were higher in depressive patients compared with patients without depression. An important concern in the relationship between hypothyroidism and depression is the etiology of hypothyroidism. In a study among patients with Hashimoto’s thyroiditis, SCH was confirmed in 85% of the participants [14], and the serum levels of TPOAb significantly correlated with depressive symptoms. However, serum levels of thyroglobulin antibodies were not associated with depression. As the TPOAb serum levels in Hashimoto’s thyroiditis correlate with the functional status of the thyroid gland, the authors suggested that impaired thyroid function rather than autoimmunity was associated with depression. Our data are consistent with the above mentioned results. Although both serum levels of TPOAb and TRAb were elevated in Graves’ patients treated with ^131^I, only TPOAb levels were closely associated with depression, while the TRAb levels were not significantly different between patients with or without depression. As TRAb are the most specific serum markers for Graves’ disease, the present study does not support a correlation between autoimmunity and depression.

The issue of whether L-thyroxine therapy should be administered to patients with SCH is controversial [15–20]. Some studies have indicated that L-thyroxine therapy may significantly improve symptoms of depression in patients with SCH, but other studies have not [9,15,21,22]. The Chinese Thyroid Disease Treatment Guidelines [5] provide no clear recommendation for the administration of L-thyroxine in response to SCH. The American Thyroid Association, the Association of Clinical Endocrinologists, and the Endocrine Society Expert Consensus [19] recommend L-thyroxine replacement as the primary treatment for patients with TSH levels exceeding 10 mIU/L. If TSH levels decrease below 10 mIU/L, TSH is monitored but no treatment is administered.

In this study, patients with serum TSH levels greater than 10 mIU/L received L-thyroxine replacement therapy. At six month follow-up, depression scores were significantly decreased, suggesting that SCH patients after ^131^I treatment of Graves’ disease with TSH > 10 mIU/L require timely treatment. However, thyroid hormone replacement was not provided to patients with TSH < 10 mIU/L because of the lack of sufficient clinical evidence and ethical considerations.

In a study reporting a 20-year follow-up [23], patients with SCH were found to have a more profound risk of developing clinical hypothyroidism compared to patients with normal thyroid function. However, the transition is slow, as only about 5% of patients with SCH develop clinical hypothyroidism within five years [24], which is similar to the prevalence among individuals with healthy thyroid function. However, ^131^I therapy was found to increase the risk for rapid development of clinical hypothyroidism, and therefore creates a possible higher risk for the development of depression in SCH patients. Thus, in these cases immediate treatment with thyroid hormone replacement therapy is beneficial.

## Conclusions

The results of our case-control study suggest that SCH following ^131^I treatment of Graves’ disease increases the incidence of depressive symptoms. The risk factors of developing depression among Graves’ patients after ^131^I administration include SCH, Graves’ eye syndrome, and high serum levels of TPOAb. In these patients, the severity of depressive symptoms was correlated with serum TSH levels. Among patients who received ^131^I treatment of Graves’ disease and subsequently developed SCH, L-thyroxine replacement therapy led to symptomatic improvement of depression in those with serum TSH levels exceeding 10 mIU/L.
